# New Method of Administering Chloroform

**Published:** 1868-12

**Authors:** 


### New Method of Administering Chloroform.

We have received the following abstract of a paper read by Dr. Rosebrugh of Toronto, Ontario, upon a new method of administering chloroform, which we publish with much pleasure, gladly calling attention to the *care* physicians should use in administering all anæsthetics. Very likely our report is too incomplete to base conclusions upon as to the claims of Dr. Rosebrugh as an originator of anything new or the feasibility of thus accurately determining the percent. of vapor administered.

The plan of dropping chloroform upon a napkin, spread over and held up a little from the face, is not new; it has been practiced more or less in Buffalo for a long time, and we have no doubt almost everywhere else. It is possible by this method to administer but small proportion of chloroform, but the exact amount would be with great difficulty determined. The percent. must depend upon the amount of air admitted, quantity of chloroform used and the waste. The number of drops of chloroform can be easily known, but the amount of waste and amount of air are not by this method accurately determined. However this may all be, the plan is an excellent one, and no doubt if followed according to Dr. Rosebrugh's directions would be sufficiently accurate and safe. Dr. Rosebrugh has an inventive turn, and if his attention is directed to the invention of an apparatus by which may be known the exact proportions of vapor and air and the quantities administered, we shall expect satisfactory results; it is possible that he has already attained this.

### Chloroform—New Method of Administering it.

At the meeting of the medical section of the Canadian Institute, held at the rooms of the Institute, on Saturday evening last, an important paper was read by Dr. Rosebrugh, on the subject of "Chloroform," which excited much interest on the part of the medical gentlemen present. The following is an abstract of that part of Dr. Rosebrugh's paper referring to the administration of chloroform:—
"It is a remarkable fact, that although medical gentlemen as a rule are very particular in weighing out carefully a dose of medicine to be administered by the mouth, the greatest possible irregularity proved in administering the vapor of chloroform; and many of the accidents from chloroform that are from time to time reported, are to be attributed to this cause alone. In order to attain the maximum degree of safety in administering chloroform, we must be guided by two principles, namely: the principle of *tolerance* and the principle of *definite dilution*. The administration should commence with an almost imperceptible quantity of the vapor. and the strength very gradually increased until the maximum strength is attained, which should never exceed 4½ per cent. This can be best attained by Dr. Clover's inhaler, which is a bag or reservoir charged with atmospheric air and 4 per cent. of chloroform vapor. Undoubtedly the inhaler is too cumbrous and too expensive to be generally adopted. In view of which Dr. Rosebrugh recommends a plan for the administration of chloroform, which he and some of his medical friends have practiced with success for years, and by which method he showed that the actual percentage of chloroform vapor that is being administered at a given time may be known. The method may be briefly described as follows: The patient is placed on his back, and one thickness of a linen napkin is placed over the face, and held 1½ inches from the mouth. The chloroform is dropped on the napkin from a two drachm vial, and a definite number of drops are administered per minute; the first minute one-third the full strength is given; two-thirds the second minute; and the full strength the third minute. The full strength is then administered for from two to six minutes, according to the degree of narcotism that is desired. To adults he usually gives about thirty drops per minute as a maximum dose; to children about eight years of age, eight drops per minute. The advantage that Dr. Rosebrugh claims for this method of administering chloroform are: 1st. Safety; 2d. Simplicity; 3d. The patients do not resist its gradual influence; 4th. The strength is known; 5th. Less chloroform is required.

At the conclusion of the reading of the paper a lively discussion followed, during which the merits of the method were fully acknowledged, and to Dr. Rosebrugh was given the credit of being the originator. The following gentlemen took part in the discussion, viz:—Drs. Thorburn, Small, Tempest, Rolph, Cumming, Sangster, Reeve, Canniff, Rosebrugh, Tempest and Winstanley.

A vote of thanks was given to Dr. Rosebrugh, and the hope expressed that the paper would be published.

